# Risk factors for poor multidrug-resistant tuberculosis treatment outcomes in Kyiv Oblast, Ukraine

**DOI:** 10.1186/s12879-017-2230-2

**Published:** 2017-02-07

**Authors:** Omowunmi Aibana, Mariya Bachmaha, Viatcheslav Krasiuk, Natasha Rybak, Timothy P. Flanigan, Vasyl Petrenko, Megan B. Murray

**Affiliations:** 1 Division of General Internal Medicine, The University of Texas Health Science Center at Houston, McGovern Medical School, Houston, TX USA; 2 Brown University School of Public Health, Providence, RI USA; 3 Department of Pulmonology, Bogomolets National Medical University, Kyiv, Ukraine; 4 Division of Infectious Diseases, Warren Alpert Medical School at Brown University, Providence, RI USA; 5 Department of Global Health and Social Medicine, Harvard Medical School, Boston, MA USA

**Keywords:** Multidrug-resistant, Tuberculosis, Risk factors, Treatment outcomes

## Abstract

**Background:**

Ukraine is among ten countries with the highest burden of multidrug- resistant TB (MDR-TB) worldwide. Treatment success rates for MDR-TB in Ukraine remain below global success rates as reported by the World Health Organization. Few studies have evaluated predictors of poor MDR-TB outcomes in Ukraine.

**Methods:**

We conducted a retrospective analysis of patients initiated on MDR-TB treatment in the Kyiv Oblast of Ukraine between January 01, 2012 and March 31st, 2015. We defined good treatment outcomes as cure or completion and categorized poor outcomes among those who died, failed treatment or defaulted. We used logistic regression analyses to identify baseline patient characteristics associated with poor MDR-TB treatment outcomes.

**Results:**

Among 360 patients, 65 (18.1%) achieved treatment cure or completion while 131 (36.4%) died, 115 (31.9%) defaulted, and 37 (10.3%) failed treatment. In the multivariate analysis, the strongest baseline predictors of poor outcomes were HIV infection without anti-retroviral therapy (ART) initiation (aOR 10.07; 95% CI 1.20–84.45; *p* 0.03) and presence of extensively-drug resistant TB (aOR 9.19; 95% CI 1.17–72.06; *p* 0.03). HIV-positive patients initiated on ART were not at increased risk of poor outcomes (aOR 1.43; 95% CI 0.58–3.54; *p* 0.44). There was no statistically significant difference in risk of poor outcomes among patients who received baseline molecular testing with Gene Xpert compared to those who were not tested (aOR 1.31; 95% CI 0.63–2.73).

**Conclusions:**

Rigorous compliance with national guidelines recommending prompt initiation of ART among HIV/TB co-infected patients and use of drug susceptibility testing results to construct treatment regimens can have a major impact on improving MDR-TB treatment outcomes in Ukraine.

**Electronic supplementary material:**

The online version of this article (doi:10.1186/s12879-017-2230-2) contains supplementary material, which is available to authorized users.

## Background

Tuberculosis (TB) control remains a major challenge for former Soviet Union countries including Ukraine, which is facing a rise in cases of multidrug-resistant TB (MDR-TB), defined as resistance to isoniazid (H) and rifampicin (R). Ukraine is currently among the ten countries with the highest MDR-TB burden worldwide, and is second only to Russia in the World Health Organization (WHO) European Region [1]. The WHO reported that 25% of newly diagnosed and 58% of retreatment TB cases in Ukraine were MDR in 2015 [2]. Treatment for MDR-TB is longer, more toxic and more expensive than for drug-sensitive TB, and WHO reports that MDR-TB treatment success rates remain low at 52% [1]. In Ukraine, the reported MDR treatment success rate is even lower with only 39% of patients treated in 2013 having good outcomes [2]. Given the burden of MDR-TB in Ukraine, there is an urgent need to develop strategies to improve treatment outcomes. A first step in this process involves identifying factors associated with poor treatment outcomes in this context.

Previous research conducted in many different contexts suggests that MDR-TB treatment outcomes vary both by factors specific to individual patients and to local TB treatment programs. Patient-level predictors of poor MDR-TB outcomes have included HIV [3–6], alcohol and substance use [6–9], smoking [10], and low body mass index [6, 7, 11, 12]. Programmatic determinants have included treatment duration, access to drug sensitivity testing and individualized treatment regimens and use of directly observed therapy (DOT) [13]. Here, we evaluate predictors of final treatment outcomes among patients initiated on MDR-TB treatment in Kyiv Oblast, Ukraine.

## Methods

### Setting and program description

We conducted a retrospective study to identify patient predictors of poor treatment outcomes among MDR-TB cases in the Kyiv Oblast of Ukraine. In this oblast, the notification rate for new pulmonary TB in 2014 was approximately 62 per 100 000 persons. Diagnosis and management of resistant TB in Kyiv Oblast is performed according to Ukraine’s National TB Program (UNTP) guidelines, which specify that diagnosis and management of TB are free of charge [14]. All patients evaluated for TB should undergo sputum smear microscopy, culture and molecular testing with Xpert® MTB/RIF; Xpert was introduced in Kyiv Oblast in December 2012 though its use was sometimes limited by availability of reagents. Cultures are performed in Löwenstein–Jensen (LJ) solid media and in BACTEC liquid media using fluorometric BACTEC MGIT960 system. For all culture positive isolates, guidelines specify baseline drug susceptibility testing (DST) to rifampin (R), isoniazid (H), ethambutol (E), pyrazinamide (Z) and streptomycin (S). Isolates that are resistant to isoniazid and rifampin are tested for resistance to second-line drugs including fluoroquinolones (ofloxacin, levofloxacin, moxifloxacin), second-line injectables (capreomycin, kanamycin, amikacin), para-aminosalicylic acid, etionamide and cycloserine. Monthly smear microscopy and culture is recommended for all patients with resistant TB, and DST is recommended every 3 to 4 months for patients who remain culture positive.

UNTP guidelines specify that all DR-TB patients should undergo at least 8 months of intensive phase treatment in a TB hospital followed by at least 12 months of continuation phase in an outpatient setting under the WHO recommended DOTS-plus strategy. The standardized intensive phase regimen includes a second-line injectable, a fluoroquinolone, pyrazinamide, protionamide or etionamide, cycloserine and para-aminosalicylic acid followed by the same drugs without an injectable during the continuation phase. Guidelines also recommend individualized treatment based on patients’ DST profiles. The UNTP specifies daily DOT during inpatient and outpatient phases of treatment. However, compliance with hospitalization is not enforced and patients can leave and return to the hospital at their own discretion. Furthermore, adherence to daily DOT varies in the outpatient clinics, where there are no dedicated TB case workers.

HIV testing is recommended for all DR-TB patients at baseline and subsequently as indicated by clinical assessment during treatment. Anti-retroviral therapy (ART) is recommended as soon as possible after initiation of TB treatment for all HIV/TB patients. ART is initiated during hospitalization for HIV/TB co-infected patients while ongoing HIV care after discharge occurs in separate HIV programs distinct from outpatient TB clinics.

In Kyiv Oblast, culture and DST are performed in the designated Biosafety Level 3 (BSL-3) laboratory facility affiliated with the region’s central TB hospital. The Kyiv Oblast laboratory performs DST using the proportion method on LJ medium for all first-line drugs, ofloxacin, capreomycin, kanamycin, etionamide, cycloserine and para-aminosalicylic acid. The M960 system is used in Kyiv Oblast to test susceptibility to pyrazinamide, levofloxacin, moxifloxacin and amikacin. DST is not performed for Linezolid, Clofazimine or Clarithromycin.

### Data collection and analysis

We analyzed routinely collected TB program data for patients 18 years and older who initiated treatment between January 01, 2012 and March 31, 2015 for MDR-TB that was confirmed by DST in Kyiv Oblast. We excluded patients who were still undergoing treatment and did not yet have a final outcome at the time of data collection. Routine program and clinical data in Kyiv Oblast is compiled in an electronic database managed by TB program staff. We extracted the following baseline demographic and clinical information from the database: age, gender, history of prior TB treatment, mode of case finding, HIV status with ART initiation date, alcohol abuse, intravenous drug use (IVDU), employment, residence, homelessness, and history of previous incarceration. We also collected baseline sputum smear, culture, Xpert and DST results and intensive phase drug regimen. We further classified patients as having extensively-drug resistant TB (XDR-TB) if they had resistance to any fluoroquinolone and at least one injectable second-line drug. We categorized drugs as effective during the intensive phase if the baseline DST demonstrated susceptibility to the drug, as ineffective if the baseline DST showed resistance and as unknown effectiveness if baseline susceptibility testing was not documented or not performed. We classified effectiveness of protionamide according to etionamide DST result and terizidone effectiveness based on cycloserine DST result.

Treatment outcomes for MDR-TB are determined according to national guidelines [14]. Patients are considered cured if they have at least 5 consecutive negative cultures performed at least 30 days apart during the last 12 months of treatment; as having completed treatment if they received the full prescribed regimen but have fewer than 5 culture results during the last 12 months of therapy; and as having failed if treatment was discontinued or if there was a change of at least 2 drugs for any of the following reasons: lack of conversion at the end of intensive phase, bacteriologic reversion to positive after initial conversion to negative results (initial conversion is based on 2 consecutive negative culture results at least 30 days apart), evidence of further acquired resistance to a fluoroquinolone or second-line injectable; or severe adverse reaction. Deaths during TB treatment are considered TB related, according to WHO guidelines [15], and detailed cause of death was not routinely recorded. A patient who interrupts treatment for 2 or more consecutive months for any reason is considered to have defaulted. We classified good treatment outcomes as treatment cure and completion and poor treatment outcomes included treatment failure, death or default. We excluded from the analysis patients who transferred out or had missing outcomes. The exact dates of treatment outcomes or last follow up visit were not available in the database.

We used Fisher’s exact test to compare categorical variables and Wilcoxon rank sum test for continuous variables. We conducted univariate and multivariate logistic regression to determine baseline factors associated with poor MDR-TB treatment outcomes. We constructed the multivariate model by including variables identified a priori as potential confounders and predictors of outcomes (age, gender, HIV status, alcohol abuse, IVDU, use of Xpert) and variables associated with poor outcome in the univariate model at *p* value < 0.2. We used complete case analysis for the regression models and excluded from the final adjusted analysis any variable for which more than 10% of patients were missing data. We considered 2-sided *p* values range between 0.02 and 0.08 as borderline significant.

## Results

We identified 617 patients initiated on MDR-TB treatment between January 01, 2012 and March 31, 2015; 239 (38.7%) of these were still on treatment at the time of data extraction and were excluded from the analysis. Table 1 lists baseline characteristics of the remaining 378 patients and distribution of treatment outcomes. The median age was 38.2 (IQR 33.0–48.6), 292 (77.2%) were male, and almost all patients (305; 84.5%) had a prior history of TB treatment. Only 6 (1.6%) patients were missing an HIV status and 82 (21.7%) were HIV-positive. Among the HIV infected, 51 (62.2%) were on ART during TB treatment. Of the 27 HIV-positive patients initiated on ART after MDR-TB diagnosis, median time from MDR treatment initiation to ART start was 42.0 days (IQR 25.0–65.0). Baseline Xpert testing was performed in 111 (29.4%) patients. Among patients enrolled after December 31, 2012 when Xpert® MTB/RIF was first mandated in the national guidelines, 38.5% (109/283) received baseline Xpert test while 59.5% (50/84) of patients enrolled after December 31, 2013 had an Xpert test. Eighteen patients (4.8%) were missing outcome data, and of the remaining 360 patients, 131 (36.4%) died, 115 (31.9%) defaulted, 37 (10.3%) failed treatment, 41 (11.4%) were cured, 24 (6.7%) completed treatment, and 12 (3.3%) transferred out.

**Table 1:** Baseline characteristics of patients initiated on multidrug-resistant tuberculosis treatment in Kyiv Oblast, Ukraine (*N* = 378)

	Cure and Completed (*N* = 65) *n* (%) or median (IQR)	Default (*N* = 115) *n* (%) or median (IQR)	Death (*N* = 131) *n* (%) or median (IQR)	Failure (*N* = 37) *n* (%) or median (IQR)	Total (*N* = 378)^a^ *n* (%) or median (IQR)
Age	36.3 (30.2–44.9)	40.2 (35.2–49.7)	38.2 (33.1–49.6)	36.7 (31.7–42.8)	38.2 (33.0–48.6)
Male	41 (63.1%)	93 (80.9%)	110 (84.0%)	30 (81.1%)	292 (77.2%)
Previous TB treatment^b^	48 (75.0%)	96 (88.1%)	104 (84.6%)	31 (83.8%)	305 (84.5%)
Passive Case Finding^c^	64 (98.5%)	110 (96.5%)	131 (100.0%)	36 (100.0%)	371 (98.7%)
HIV positive^d^	11 (17.2%)	17 (15.0%)	43 (33.1)	5 (13.5%)	82 (21.7%)
On ART during MDR-TB treatment among HIV positive	10 (90.9%)	12 (70.6%)	25 (58.1%)	1 (20.0%)	51 (62.2%)
Smear positive at baseline^e^	25 (38.5%)	64 (55.7%)	88 (67.2%)	25 (69.4%)	220 (58.4%)
Alcohol abuse	11 (16.9%)	25 (21.7%)	27 (20.6%)	4 (10.8%)	73 (19.3%)
IVDU	2 (3.1%)	0 (0.0%)	6 (4.6%)	2 (5.4%)	11 (2.9%)
Unemployed	30 (46.2%)	75 (65.2%)	87 (66.4%)	22 (59.5%)	235 (62.2%)
Homeless	0 (0.0%)	4 (3.5%)	7 (5.3%)	1 (2.7%)	13 (3.4%)
Rural Residence^f^	35 (58.3%)	33 (37.1%)	56 (53.3%)	14 (42.4%)	162 (51.8%)
Medical Worker in TB	1 (1.5%)	0 (0.0%)	0 (0.0%)	0 (0.0%)	2 (0.5%)
Previous Incarceration	2 (3.1%)	4 (3.5%)	7 (5.4%)	1 (2.7%)	15 (4.0%)
Known TB contact	1 (1.5%)	1 (0.9%)	1 (0.8%)	0 (0.0%)	3 (0.8%)
Baseline Gene Xpert Test	15 (23.1%)	34 (29.6%)	39 (29.8%)	9 (24.3%)	111 (29.4%)
Extensively drug-resistant (XDR-TB)	2 (3.1%)	6 (5.2%)	18 (13.7%)	5 (13.5%)	35 (9.3%)

*IQR* interquartile range, *ART* anti-retroviral therapy, *IVDU* intravenous drug use

^a^Total N includes patients with unknown outcome and those who transferred out

^b^Missing observations, *N* = 17

^c^Missing observations, *N* = 2

^d^Missing observations, *N* = 6

^e^Missing observations, *N* = 1

^f^Missing observations, *N* = 65

The median duration of intensive phase treatment was 236.1 days (IQR 144.9–240.0 days). Only 15 (4.0%) patients received at least four intensive phase drugs to which their baseline isolates were susceptible, and 235 (62.7%) received at least one drug during the intensive phase to which their baseline isolates were known to be resistant. Two hundred and thirty-eight patients (63.5%) received at least four intensive phase drugs for which their baseline isolates did not have drug susceptibility data.

In the univariate analysis, patients were more likely to have poor outcomes if they were male (OR 2.73; 95% CI 1.51–4.92; *p* 0.001), had a previous TB history (OR 2.03; 95% CI 1.05–3.93; *p* 0.04), were smear positive (OR 2.70; 95% CI 1.55–4.70; *p* 0.001), or unemployed (OR 2.17; 95% CI 1.26–3.74; *p* 0.01) [Table 2]. Compared to HIV-negative patients, HIV-positive patients who were not initiated on ART had borderline increased risk of poor outcomes (OR 6.65; 95% CI 0.88–50.07; *p* 0.07) while those who were infected and received ART were not at increased risk (OR 0.94; 95% CI 0.44–2.00; *p* 0.87).

**Table 2:** Univariate and multivariate analyses of baseline predictors of poor multidrug-resistant tuberculosis treatment outcomes

	Univariate Analysis (*N* = 348)	*p* value	Multivariate Analysis (*N* = 328)	*p* value
	OR (95% CI)		OR (95% CI)	
Age	1.01 (0.99–1.04)	0.35	1.02 (0.99–1.04)	0.24
Male	2.73 (1.51–4.92)	0.001	1.86 (0.96–3.64)	0.07
Previous TB treatment^a^	2.03 (1.05–3.93)	0.04	2.29 (1.06–4.94)	0.03
Passive Case Finding^b^	1.08 (0.12–9.85)	0.94	NA	
HIV negative^c^	Ref		Ref	
HIV positive with ART	0.94 (0.44–2.00)	0.87	1.43 (0.58–3.54)	0.44
HIV positive without ART	6.65 (0.88–50.07)	0.07	10.07 (1.20–84.45)	0.03
Smear Positive^d^	2.70 (1.55–4.70)	0.001	2.54 (1.37–4.70)	0.003
Alcohol	1.21 (0.60–2.47)	0.60	0.96 (0.42–2.22)	0.93
IVDU	0.92 (0.19–4.42)	0.91	0.26 (0.04–1.87)	0.18
Unemployed	2.17 (1.26–3.74)	0.01	1.97 (1.03–3.78)	0.04
Rural Residence^e^	1.69 (0.95–3.00)	0.08	NA	
Previous Incarceration	1.39 (0.30–6.39)	0.67	NA	
Known TB contact	0.46 (0.04–5.10)	0.52	NA	
Baseline Gene Xpert Test	1.36 (0.72–2.56)	0.34	1.31 (0.63–2.73)	0.47
XDR-TB	3.60 (0.84–15.47)	0.09	9.19 (1.17–72.06)	0.03

Complete case analysis used in logistic regression

*OR* odds ratio, *CI* Confidence Interval, *ART* anti-retroviral therapy, *IVDU* intravenous drug use, *XDR* extensively drug-resistant

^a^Missing observations, *N* = 15

^b^Missing observations, *N* = 2

^c^Missing observations, *N* = 4

^d^Missing observations, *N* = 1

^e^Missing observations, *N* = 61; excluded from multivariate analysis because > 10% of data missing

After adjusting for potential confounders, the following characteristics remained predictors of poor treatment outcomes: history of TB treatment (aOR 2.29; 95% CI 1.06–4.94; *p* 0.03), HIV-positive without ART initiation (aOR 10.07; 95% CI 1.20–84.45; *p* 0.03), smear positivity (aOR 2.54; 95% CI 1.37–4.70; *p* 0.003), unemployment (aOR 1.97; 95% CI 1.03–3.78; *p* 0.04) and XDR-TB (aOR 9.19; 95% CI 1.17–72.06; *p* 0.03) [Table 2]. Male patients were also more likely to experience poor outcomes but this result was of borderline significance after adjustment (aOR 1.86; 95% CI 0.96–3.64; *p* 0.07). In the adjusted analysis, there was no statistically significant difference in risk of poor outcomes among those with baseline Xpert testing compared to those who did not receive Xpert (aOR 1.31; 95% CI 0.63–2.73).

## Discussion

We found only 18% of patients treated for MDR-TB achieved treatment cure or completion in Kyiv Oblast, Ukraine. The strongest baseline predictors of poor outcomes included lack of ART among HIV-positive patients and presence of XDR-TB, which conferred 10- and 9-fold increases in risk respectively. We did not detect a statistically significant association between the use of Xpert and MDR treatment outcomes.

This study found lower rates of MDR treatment success than had been previously reported in other former Soviet Union countries where success rates range from 34% in a region of Belarus [16] and 45% in 8 districts of Moldova [17] to 68% in a national cohort in Latvia [18] and 77% in Tomsk Oblast of Russia [19]. Our findings are however similar to the only published study assessing risk factors for poor MDR-TB outcomes in Ukraine [20]. In that cohort of 484 patients treated between 2006 and 2011 in Kyiv city, Lytvynenko et al. [20] found that only 22% of patients achieved treatment success and identified bilateral lung involvement, previous DR-TB treatment, poor adherence and XDR-TB as predictors of poor outcomes. Of note, this study reported a much lower HIV prevalence (3%) than in our cohort, and more than one-third of patients were missing final treatment outcomes; the authors only assessed risk factors for poor outcomes during the intensive phase of treatment.

Our analysis identified a number of ways that treatment outcomes could be improved in this setting. Although our cohort included a small number of HIV-positive patients who were not on ART, we found that this group of patients were at very high risk of poor MDR-TB treatment outcomes while those who did receive ART experienced similar outcomes to non-HIV infected patients. This finding is consistent with the results of multiple previous studies that have shown that the use of ART in HIV-positive patients with resistant tuberculosis markedly improves outcomes [11, 12, 21, 22]. Although HIV status was ascertained in almost all the patients in our cohort, 38% of the HIV infected did not receive ART during TB treatment despite national guidelines that recommend prompt initiation of ART for all co-infected patients. We did not have data on specific reasons for limited ART initiation; anecdotal reports suggest some providers deferred ART for patients with high CD4 counts despite UNTP recommendation for ART regardless of CD4 count. This implies there are barriers to universal ART coverage for co-infected patients, and these should be addressed since careful compliance with national guidelines could markedly improve outcomes among these patients.

Secondly, the WHO recommends that MDR-TB treatment regimens should include at least four second-line drugs that are likely to be effective in addition to pyrazinamide [15] and studies show that regimens containing more than four effective drugs further reduce the risk of death or failure [23, 24] and recurrence [25]. Although the UNTP does not specify number of likely effective drugs that should be included MDR-TB regimens, the guidelines do recommend that patients receive individualized treatments based on DSTs. However, despite the existence of laboratory capacity to perform DST to all first line and many second line drugs, the majority of the patients in our cohort received drugs, which were either ineffective or were not known to be effective because DSTs were not performed or not readily available to the clinician sometimes due to delays in transferring laboratory results into the clinical database used by TB providers. Fewer than 5% of these patients received at least four likely effective drugs during the intensive phase of treatment. This underscores a need to understand and address programmatic barriers that interfere with performing DST even when these tests are available as well as the barriers that prevent providers from utilizing available DST results to minimize use of ineffective drugs.

Finally, although the use of Gene Xpert to identify patients with drug resistant TB increased over calendar time, about 40% of patients had not been evaluated with Xpert one year after it had been mandated by the UNTP. In principle, since Xpert enables earlier detection of drug resistance, it should reduce the time to initiation of optimal therapy and thereby improve outcomes. However, here we found that the outcomes of patients who had received Xpert testing at baseline were no different from those who had not received the test. These results are similar to the findings from a recent study from South Africa [26], which showed that the use of Xpert did not improve treatment outcomes for MDR-TB. Thus, it is not clear that compliance with this guideline would have resulted in better outcomes for the patients in our cohort.

Our study has several limitations. First, we analyzed routine program data, which did not include a comprehensive assessment of other patient risk factors (e.g. smoking, nutritional status, diabetes) associated with poor TB outcomes. Second, UNTP does not employ validated screening tools and thus likely underestimates prevalence of alcohol and substance abuse, which are important determinants of MDR-TB treatment outcomes. We also did not specifically evaluate treatment adherence. In this setting, factors that limit adherence likely include lack of rigorous adverse event monitoring, limited access to concurrent treatment for alcohol and substance abuse and the requirement for prolonged hospitalizations; anecdotal reports suggest that patients often leave and return to the hospital at their discretion.

Despite these limitations, our findings suggest that rigorous compliance with existing UNTP guidelines regarding ART for HIV infected patients and individualized treatment regimens based on DSTs will have a major impact on improving MDR treatment outcomes. Programs in other settings have also successfully reduced poor MDR outcomes by addressing social risk factors that limit treatment adherence such as providing material incentives in the form of food, cash or transportation, providing psychosocial support and using community health workers to provide DOT [27–29]. Ukraine may also benefit from adapting such patient-centered strategies for the national TB program.

## Conclusion

We found extremely low rates of treatment success for multidrug-resistant TB in Kyiv Oblast, Ukraine. This highlights an urgent need to address challenges in the country’s TB program in order to successfully combat the drug resistant TB epidemic.

## Additional file

Additional file 1: Study data. Dataset for cohort of patients initiated on treatment for multi-drug resistant tuberculosis between January 1, 2012 and March 31, 2015 in Kyiv Oblast, Ukraine. (XLSX 38 kb)

## Abbreviations

ART: Anti-retroviral therapy
BSL: Biosafety Level
CI: Confidence Interval
DOT: Directly observed therapy
DST: Drug-susceptibility testing
IQR: Interquartile range
IVDU: Intravenous drug use
LJ: Löwenstein–Jensen
MDR: Multidrug-resistant
OR: Odds ratio
TB: Tuberculosis
UNTP: Ukraine’s National TB Program
WHO: World Health Organization
XDR: Extensively drug-resistant
